# Examining the Effects of Nutrient Supplementation on Metabolic Pathways via Mitochondrial Ferredoxin in Aging Ovaries

**DOI:** 10.3390/nu16101470

**Published:** 2024-05-13

**Authors:** Chia-Chun Wu, Chia-Jung Li, Li-Te Lin, Zhi-Hong Wen, Jiin-Tsuey Cheng, Kuan-Hao Tsui

**Affiliations:** 1 Department of Biological Sciences, National Sun Yat-sen University, Kaohsiung 804, Taiwan; michellewu0214@gmail.com; 2 Department of Obstetrics and Gynaecology, Kaohsiung Veterans General Hospital, Kaohsiung 813, Taiwan; nigel6761@gmail.com (C.-J.L.); litelin1982@gmail.com (L.-T.L.); 3 Institute of Biopharmaceutical Sciences, National Sun Yat-sen University, Kaohsiung 804, Taiwan; 4 Department of Obstetrics and Gynaecology, National Yang-Ming University School of Medicine, Taipei 112, Taiwan; 5 Department of Marine Biotechnology and Resources, National Sun Yat-sen University, Kaohsiung 804, Taiwan; wzh@mail.nsysu.edu.tw; 6 Department of Obstetrics and Gynecology, Taipei Veterans General Hospital, Taipei 112, Taiwan; 7 Department of Medicine, Tri-Service General Hospital, National Defense Medical Center, Taipei 114, Taiwan

**Keywords:** multi-omics, nutrients, FDX1, ovarian aging

## Abstract

As women age, oocytes are susceptible to a myriad of dysfunctions, including mitochondrial dysfunction, impaired DNA repair mechanisms, epigenetic alterations, and metabolic disturbances, culminating in reduced fertility rates among older individuals. Ferredoxin (FDX) represents a highly conserved iron–sulfur (Fe–S) protein essential for electron transport across multiple metabolic pathways. Mammalian mitochondria house two distinct ferredoxins, FDX1 and FDX2, which share structural similarities and yet perform unique functions. In our investigation into the regulatory mechanisms governing ovarian aging, we employed a comprehensive multi-omics analysis approach, integrating spatial transcriptomics, single-cell RNA sequencing, human ovarian pathology, and clinical biopsy data. Previous studies have highlighted intricate interactions involving excessive lipid peroxide accumulation, redox-induced metal ion buildup, and alterations in cellular energy metabolism observed in aging cells. Through a multi-omics analysis, we observed a notable decline in the expression of the critical gene FDX1 as ovarian age progressed. This observation prompted speculation regarding FDX1’s potential as a promising biomarker for ovarian aging. Following this, we initiated a clinical trial involving 70 patients with aging ovaries. These patients were administered oral nutritional supplements consisting of DHEA, ubiquinol CoQ10, and Cleo-20 T3 for a period of two months to evaluate alterations in energy metabolism regulated by FDX1. Our results demonstrated a significant elevation in FDX1 levels among participants receiving nutritional supplementation. We hypothesize that these nutrients potentiate mitochondrial tricarboxylic acid cycle (TCA) activity or electron transport chain (ETC) efficiency, thereby augmenting FDX1 expression, an essential electron carrier in metabolic pathways, while concurrently mitigating lipid peroxide accumulation and cellular apoptosis. In summary, our findings underscore the potential of nutritional intervention to enhance in vitro fertilization outcomes in senescent cells by bolstering electron transport proteins, thus optimizing energy metabolism and improving oocyte quality in aging women.

## 1. Introduction

Ovarian aging, a natural and physiological process, entails the gradual decline in both the quantity and quality of oocytes and follicles. While aging is an inevitable aspect of human life, the ovaries exhibit signs of aging early on, typically around the age of 35, leading to age-related infertility [1]. This decline is attributed to factors such as a reduced number of primordial follicles (PMFs) at birth and increased depletion during the reproductive years. The consequences of ovarian aging include diminished reproductive capacity, infertility, and the onset of clinical symptoms like endocrine imbalances and irregular menstrual cycles [2].

In recent years, there has been a burgeoning interest in exploring the potential of nutritional supplements to alleviate the impacts of ovarian aging [3]. These supplements encompass a range of nutrients, including antioxidants, vitamins, minerals, and phytochemicals, which have garnered attention for their ability to combat oxidative stress, dampen inflammation, and bolster overall ovarian well-being. By offering a non-invasive and readily accessible approach, these supplements hold promise for enhancing fertility outcomes among aging women [4,5]. Nutritional supplementation emerges as a compelling strategy for addressing the age-related shifts in ovarian function and fertility. By specifically targeting oxidative stress, inflammation, and gut health, these supplements have the potential to fortify ovarian health and elevate reproductive success in women grappling with ovarian aging [6]. Further investigations in this realm are essential to unraveling the intricate mechanisms at play and refining supplementation protocols to yield maximal benefits.

This study employed a multi-omics analysis to assess the potential of FDX1 as a biomarker for ovarian aging. Additionally, we employed a nutrigenomics approach to validate ovarian aging in women with poor ovarian response (POR) who were administered nutritional supplements (dehydroepiandrosterone, DHEA). Furthermore, we investigated the effects of androsterone (DHEA), coenzyme Q10 (CoQ10), and triiodothyronine (T3) on ovarian quality. DHEA, predominantly synthesized by the adrenal glands but also by the gonads and brain, represents a primary steroid hormone circulating throughout the human body [7]. It is chiefly produced by cumulus-granulosa cells within ovarian follicles, serving as a pivotal female hormone [8]. CoQ10 (CoQ10), an essential constituent of aerobic respiration found ubiquitously in eukaryotes, plays a central role in the electron transport chain and mitochondrial respiration [9]. CoQ10 is present throughout the body in cell membranes, particularly in mitochondrial membranes, and is predominantly abundant in the heart, lungs, liver, kidneys, spleen, pancreas, and adrenal glands. The total body content of CoQ10 is estimated to be only about 500–1500 mg and declines with age [10]. T3, a thyroid hormone, governs a myriad of physiological processes encompassing growth, metabolism, body temperature, and heart rate [11]. With swifter and more potent physiological effects compared to thyroxine (T4), approximately 20% of T3 is directly secreted and synthesized by the thyroid gland, while the remaining 80% is converted from T4 in circulation [12]. T3’s synthesis and secretion are stimulated by the thyroid-stimulating hormone (TSH) secreted by the pituitary gland, rendering it more biologically active than T4, despite its lower serum levels [13].

Ferredoxin, a protein characterized by its low molecular weight (184 a.a.) and negative charge under neutral pH, plays a pivotal role in cellular redox processes. This protein features iron–sulfur clusters, facilitating electron transfer from nicotinamide adenine dinucleotide phosphate (NADPH) via ferredoxin reductase (FDXR) to various cellular systems involved in fundamental biological processes [14]. Among humans, the mitochondrial ferredoxins FDX1 and FDX2 are of particular importance, with distinct functions in key metabolic pathways [15]. FDX1, encoded by the ferredoxin 1 gene, acts as an essential mediator in steroidogenesis, heme biosynthesis, and Fe–S cluster formation. Its role in transferring electrons to mitochondrial cytochrome P450 via ferredoxin reductase is critical for the metabolic pathways governing steroid hormones, vitamin D metabolism, and bile acid production [16]. Additionally, recent research has uncovered FDX1’s involvement in cuproptosis regulation and protein lipidation processes, highlighting its broader impact on cellular homeostasis and signaling networks. Within mammalian cells, both FDX1 and FDX2 localize to mitochondria, underscoring their importance in maintaining mitochondrial function and metabolic homeostasis [17].

This study aimed to assess the potential benefits of nutritional supplements in modulating genes responsible for mitochondrial metabolism in aging germ cells. By employing spatial transcriptomics on young and aged mouse ovaries, we sought to elucidate the significance of the gene FDX1 within the ovarian context. Through an enrichment analysis of intercellular interactions and regulatory mechanisms across different ovarian cell types, we aimed to identify targetable genes for intervention. Furthermore, the study investigated the impact of nutritional supplements on these genes in infertile and aging germ cells. Employing a multi-omics approach, we screened various nutritional supplements and assessed their effectiveness in patients with aging ovaries. Additionally, we examined the supplements’ influence on cellular energy metabolism to mitigate age-related cellular damage. These findings offer a novel strategy and diagnostic marker for addressing ovarian aging.

## 2. Materials and Methods

### 2.1. Spatial Transcriptomics Analysis of Mouse Ovaries

This study employed spatial transcriptomics analysis to delve deeper into the expression pattern and spatial organization of FDX1 in sections of mouse ovarian tissue. Females were naturally aged to represent young (Young; 3–4 months old, n = 4) and aging (Aged; 15–16 months old, n = 4) groups. Data from a prior study (GSE188257) were utilized, focusing on ovaries from both young and aged female mice. Initially, unique molecular identifiers (UMIs) within each spot were amalgamated and categorized into bins delineated by bin100. Clusters were then annotated based on hematoxylin and eosin (H&E) staining, ensuring data integrity by excluding spots with aberrant characteristics, such as those containing over 10% mitochondrial genes or fewer than 200 detected gene counts. Subsequently, further annotation was meticulously conducted using cell markers to complete the dataset. Employing advanced dimensionality reduction techniques like the Run-PCA function, followed by the FindNeighbors and FindClusters functions, facilitated the clustering of similar spatial transcriptome points. This strategic clustering method aided in identifying common cell types by utilizing an average representation matrix of different clusters, providing a nuanced comprehension of spatial transcriptome dynamics [18].

### 2.2. Ethics Statement

Ethical clearance for this study was obtained from the Institutional Review Board of Kaohsiung Veterans General Hospital (Approval ID: KSVGH21-CT8-13, 16 September 2022 to 15 September 2023). Adherence to the ethical principles outlined in the Declaration of Helsinki ensured protection of the participants’ welfare, rights, and privacy. The review process involved a comprehensive assessment of the study protocol, including considerations for participant recruitment, informed consent procedures, data handling, and confidentiality measures. This rigorous ethical oversight underscores our commitment to upholding the highest standards of research integrity and participant protection throughout the duration of the study.

### 2.3. Clinical Biopies and Collection

**Figure 1:**
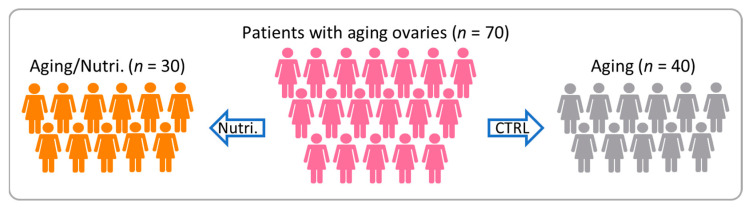
The flowchart serves as a visual representation of the systematic approach followed in conducting the research and ensuring the selection of appropriate studies for inclusion in the analysis.

In May 2021, the in vitro fertilization (IVF) Center at Kaohsiung Veterans General Hospital embarked on a study aimed at investigating the efficacy of nutritional supplements in infertile patients. The study involved the recruitment of participants divided into two groups: an aging group and an aging group supplemented with nutritional interventions (aging/nutri.). Patients in the nutritional supplement group were administered daily doses of DHEA capsules (25 mg, New Dios-25 Veg capsules by Toppure Biotechnology Co., Ltd., Taipei, Taiwan), ubiquinol CoQ10 capsules (30 mg, Circuform Softgel capsules by Toppure Biotechnology Co., Ltd., Taipei, Taiwan), and Cleo-20 T3 soft capsules (300 mg, Supremevitamin Biotechnology, Co., Ltd., Taichung, Taiwan) for a minimum duration of 8 weeks before undergoing IVF (Figure 1). Exclusion criteria included a history of oophorectomy, prior donor cycles, pelvic radiotherapy or chemotherapy, and hormone therapy within the past 3 months. Detailed clinical characteristics of the participants, such as age, body mass index (BMI), duration of infertility, previous IVF failure, types of infertility, and basal hormone levels, were recorded and are presented in Table 1 and Table 2.

### 2.4. Human Cumulus Cell (CC) Isolation from Patients

CCs were obtained from patients following established protocols [19]. The procedure entailed isolating CCs from oocytes, mechanically dissociating them, and consolidating them into a pooled sample. The pooled CCs underwent several washes in a phosphate-buffered saline (PBS) solution containing bovine serum albumin (BSA) through centrifugation at 800× *g* for 5 min. The resulting pellet was then suspended in Histopaque 1077, supplemented with fetal bovine serum, insulin, transferrin, sodium selenite, and androstenedione. Following this, the CCs were cultured on a multiplate and placed in a humidified atmosphere with 5% CO_2_ at 37.5 °C for 24 h to facilitate further analysis. 

### 2.5. RNA Extraction and Real-Time Polymerase Chain Reaction (PCR)

RNA extraction was carried out using REzol (Protech Technology, Taipei, Taiwan), a reliable extraction reagent known for its efficiency in isolating RNA from various sample types. Following extraction, mRNA expression levels were quantified using SYBR Green-based quantitative real-time PCR (qRT-PCR, Thermo Fisher Scientific, Pleasanton, CA, USA) assays, performed on StepOne equipment manufactured by Applied Biosystems (San Francisco, CA, USA), a trusted platform for accurate and reproducible gene expression analysis. To ensure precise normalization of expression data, β-actin and RNU6-1 were utilized as reference genes, providing reliable internal controls for the qRT-PCR analysis.

### 2.6. Statistical Analysis

Measurements were repeated independently at least four times, with results presented as a mean ± S.D. Statistical significance was determined using GraphPad Prism 8.0, applying Tukey post-hoc tests after a two-way analysis of variance. A *p*-value < 0.05 was considered significant. 

## 3. Results

### 3.1. Demographic and Clinical Characteristics of Infertile Patients

To assess the impact of nutritional supplements, a cohort of 70 infertile elderly patients was recruited from the IVF Center of Kaohsiung Veterans General Hospital, segregated into two distinct groups: the aging group and the aging/nutritional supplement group. Table 1 offers comprehensive insights into the baseline characteristics of each group, encompassing factors such as age, body mass index (BMI), duration of infertility, history of previous IVF failures, types of infertility, and clinical parameters including basal FSH, basal E2, and basal LH levels. This detailed profiling enables a thorough comparison between the groups, facilitating a comprehensive analysis of their respective demographics and clinical attributes.

### 3.2. Clinical and Cyclic Characteristics of Infertile Patients

In this comprehensive analysis, we delved into the patient demographics and pregnancy outcomes derived from IVF cycles conducted within both the aging and aging/nutritional supplement groups. A thorough examination of various parameters was conducted to elucidate potential differences between the two cohorts (Table 2). Across the aging and aging/nutritional supplement groups, variables such as stimulation duration and gonadotropin dosage were meticulously assessed to gauge their impact on treatment outcomes. Furthermore, detailed scrutiny was applied to clinical features unique to each group, including the number of retrieved oocytes (6.5 ± 3.9 vs. 14.2 ± 6.4), metaphase II oocytes (5.4 ± 3.1 vs. 11.6 ± 5.2), maturation rate (79.2 ± 18.6 vs. 82.4 ± 18.2), as well as the fertilization rate (85.2 ± 20.7 vs. 84.6 ± 17.4). The administration of these nutrients effectively boosts the quantity of oocytes retrieved, metaphase II oocytes, and fertilized oocytes.

### 3.3. Spatial Transcriptomics Uncovers Cell Cluster Distinctions in Young and Old Mouse Ovaries

Spatial transcriptomics allows for the precise mapping of gene expression patterns within tissue sections, offering insights into the spatially resolved molecular signatures linked with ovarian aging. While we have previously published potential biomarkers for ovarian aging, it is noteworthy that the data presented here were cross-validated using different ovarian samples, enhancing the robustness of the findings [20]. This approach unveils spatial distribution changes in gene expression, cellular interactions, and tissue architecture alterations during aging.

**Figure 2:**
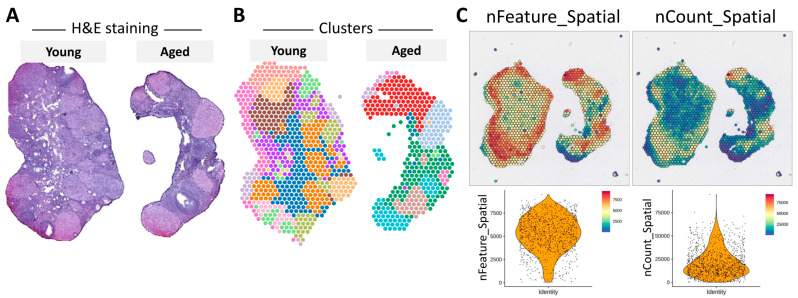
Spatial transcriptomics reveals characteristics of distinct cell clusters in young and aged mouse ovaries. Spatial transcriptomics permits in-depth examination of tissue sections, facilitating the identification and precise alignment of clusters. This method also offers insights into morphological features and diverse cell clusters, as evidenced by their correlation with H&E staining (**A**). Each color in the representation signifies a distinct cell cluster (**B**). (**C**) The dataset includes essential features such as total counts and gene counts, providing crucial information for comprehensive analysis and interpretation.

In our study, spatial transcriptomics technology was employed to map transcriptome features onto H&E-stained histological images of mouse ovaries. The analysis of these images revealed notable differences in the size and quantity of corpus luteum between aged and young mouse ovaries (Figure 2A). Through a Space Ranger analysis, we analyzed a total of 1193 cells, 4263 spots, and 32,285 measured genes, resulting in the identification of 16 distinct cell clusters in menstrual and aging ovaries (Figure 2B). During spatial transcription, it is imperative to eliminate low-quality signals to minimize technical noise and optimize subsequent signal analysis, including biological signals. This process entails filtering out genes lacking signals in any cell, cells with sparse reads, and genes displaying undetectable or extremely low expression levels. Notably, the sequencing quality chart demonstrates comparable quality between the number of genes expressed by the cells and the total number of UMIs, thereby enhancing confidence in subsequent gene screening and analysis. To ensure the quality of both cells and genes, violin plots were utilized to visualize the distribution of gene expressions normalized by gene counts and total counts (Figure 2C). This comprehensive approach not only advances our understanding of ovarian aging but also facilitates the identification of potential biomarkers with translational relevance for assessing ovarian health in humans.

**Figure 3:**
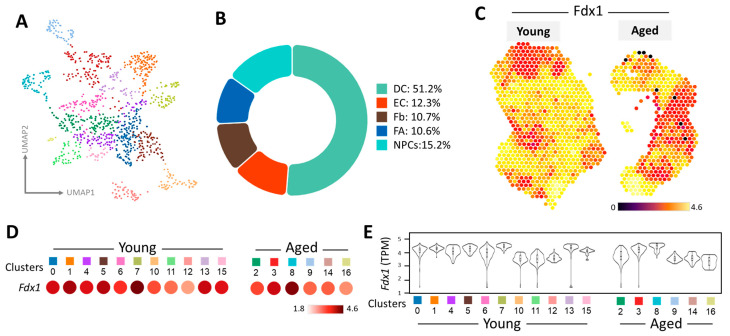
Spatial transcriptomics unveils genetic changes in FDX1 within the mouse ovary. (**A**) UMAP visualization showcases cells from young and aged ovaries, with color codes representing predominant cell types. (**B**) A pie chart illustrates the distribution of different cell populations within the ovarian microenvironment. (**C**) The heat map displays the gene expression levels and spatial distribution of FDX1 across ovaries from mice of varying ages. (**D**) Dot plots illustrate the expression patterns of FDX1 across distinct clusters. (**E**) Violin plots depict FDX1 transcript abundance in ovarian regions of young and aged mice.

We utilized Uniform Manifold Approximation and Projection (UMAP) to visualize cell clusters obtained from a Space Ranger analysis, as depicted in Figure 3A. This approach provided insights into the spatial organization of various cell types within the dataset. The accompanying pie chart in Figure 3B offers a comprehensive breakdown of the diverse cell populations present across all spatial transcriptomes, revealing their relative abundance and distribution within the ovarian microenvironment. Moving forward, Figure 3C explores the expression pattern and spatial arrangement of the mitochondrial metabolism gene FDX1, shedding light on its role in the context of ovarian aging. The thermal image illustrates that half of the aging ovaries exhibit orange–red and yellow hues. In contrast, 80% of young ovaries display a yellow signal, indicating differential transcription of FDX1 between the two age groups. Further analysis involved the classification of 16 distinct cell clusters, facilitating a comparative evaluation between younger and older groups. This comparison unveiled significant heatmap changes indicative of age-related alterations within ovarian tissue, with reduced representation observed in the aging group across various cell clusters, as illustrated in Figure 3D. These findings are corroborated by the transcripts per million (TPM) analysis depicted in Figure 3E, while the violin plot in 3E offers a clearer visualization of diminished expression levels in specific cell clusters (clusters 9, 14, and 16).

### 3.4. Analysis of Intercellular Communication Networks Reveals Insights into the Ovarian Microenvironment

As ovarian function declines with age, changes occur in the microenvironment within the ovary, including alterations in angiogenic factors and blood vessel density. Furthermore, dysfunction or dysregulation of FDX1 could impact mitochondrial function, which, in turn, might affect the production of reactive oxygen species (ROS) and other signaling molecules involved in angiogenesis. Mitochondrial dysfunction is implicated in the aging process and might contribute to the decline in ovarian function observed with aging.

**Figure 4:**
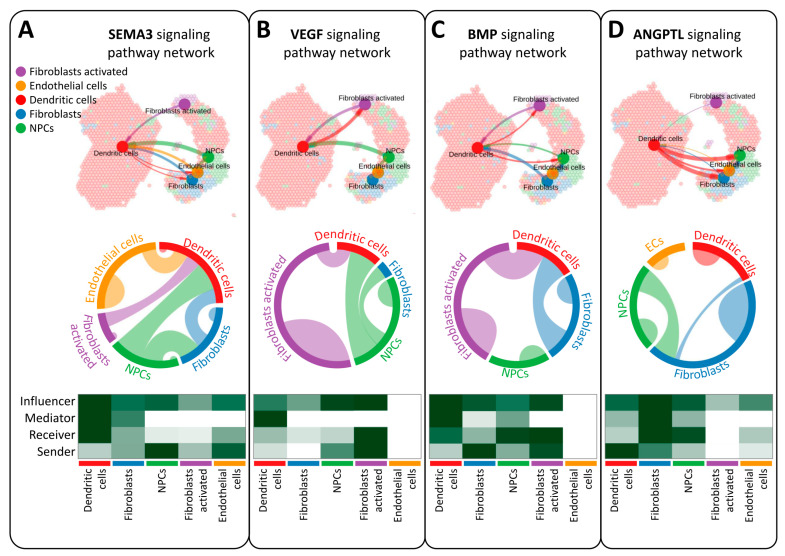
CellChat investigates intercellular communication networks among different cell types. Spatial transcriptomics identifies intercellular communication within ovarian tissue and evaluates variations in signaling pathways related to SEMA3 (**A**), VEGF (**B**), BMP (**C**), ANGPTL (**D**) and, across different cell types, along with network centrality scores.

CellChat employs sophisticated pattern recognition algorithms to quantitatively analyze ligand–receptor interactions, providing valuable insights into the intricate network of cellular communication. By predicting key incoming and outgoing signals for specific cell types, CellChat elucidates the distinct roles played by different cell groups in signal transmission pathways, including Receivers, Senders, Mediators, and Influencers. In the context of semaphorin 3E (SEMA3), vascular endothelial growth factor (VEGF), bone morphogenetic protein (BMP), and angiopoietin-like protein (ANGPTL) signaling pathways (Figure 4A–D), five cell types emerge as potential targets: activated fibroblasts, endothelial cells, dendritic cells, fibroblasts, and neural progenitor cells (NPCs). Notably, dendritic cells exhibit robust signal transmission across multiple roles in the SEMA3 pathway, underscoring their significance in intercellular communication. In contrast, endothelial cells display no signal transmission in the VEGF and BMP pathways, indicating a potential regulatory role in these processes. Similarly, activated fibroblasts do not participate in signal transmission within the ANGPTL pathway. These findings highlight the intricate nature of ligand–receptor-mediated communication among different cell types, offering valuable insights into the mechanisms underlying ovarian aging progression.

### 3.5. Nutritional Supplements Affect Changes in Cellular Metabolic Pathways

Previously, we conducted an extensive examination of FDX1 transcript levels in human ovaries, leveraging data from the Human Protein Atlas (HPA) public repository. Additionally, we recruited seventy-five patients undergoing in vitro fertilization (IVF) treatment at the IVF Center of Kaohsiung Veterans General Hospital. Our findings remain consistent across both the HPA database predictions and clinical patient genetic data. Notably, we observed a significant reduction in FDX1 expression levels in the group with aging ovaries (>37 years) compared to the group with young ovaries (<36 years) [20].

**Figure 5:**
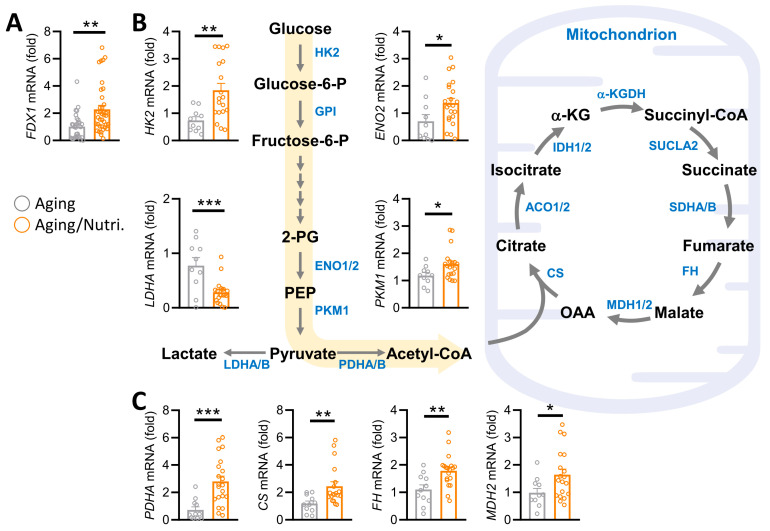
Supplementation influences the metabolic reprogramming of cumulus cells in aging ovarian patients. (**A**) QPCR analysis of FDX1 gene levels. Metabolic pathway diagram of glycolysis (**B**) and TCA cycle (**C**). * *p* < 0.05, ** *p* < 0.01, and *** *p* < 0.001.

Subsequently, we identified FDX1 as a prospective indicator of ovarian aging and delved into the impact of nutritional supplements on the molecular pathways involving FDX1 and energy metabolism genes within cumulus cells of aging patients. Our investigation unveiled a notable elevation in FDX1 gene expression following nutritional supplementation compared to the control group (Figure 5A). Furthermore, scrutiny of metabolic genes associated with glycolysis and the tricarboxylic acid (TCA) cycle exhibited significant upregulation of hexokinase 2 (HK2), enolase 2 (ENO2), and pyruvate kinase M1 (PKM1), accompanied by downregulation in the aging/nutritional group (Figure 5B). Additionally, pivotal genes governing the TCA cycle, including pyruvate dehydrogenase E1 subunit α 1 (PDHA1), citrate synthase (CS), fumarate hydratase (FH), and malate dehydrogenase 2 (MDH2), showcased alterations in expression levels (Figure 5C). These findings suggest that supplementation in older patients elicits modifications in glucose metabolism and the TCA cycle within cumulus cells, potentially bolstering cellular energy production and augmenting the success of IVF cycles. In summary, our study highlights the potential advantages of metabolic pathway modification through supplementation to improve IVF outcomes in older patients.

## 4. Discussion

Mitochondria play a pivotal role in regulating apoptosis, with apoptotic factors and damage regulation pathways exerting influence on these organelles [21]. Moreover, mitochondria are intricately linked to cellular energy metabolism [22,23]. FDX1, belonging to the iron–sulfur protein family, participates in various biological processes such as follicle development, ovulation, and the synergistic action of diverse substances [24]. The apoptosis, hormone synthesis, and luteinizing functions of granulosa cells are paramount for oocyte and embryo quality, as well as pregnancy outcomes [25]. Given that oocyte quality significantly impacts successful fertilization and implantation, employing an in vivo oocyte maturation model holds considerable value in assessing potential markers associated with oocyte quality and enhancing the in vitro maturation (IVM) culture system [26,27].

FDX1 is widely expressed in tissues involved in steroidogenesis, including the adrenal glands and gonads [28,29]. In the context of steroid synthesis, the steroid synthesis acute regulatory protein (Star) facilitates the transfer of intracytoplasmic cholesterol to mitochondria, where the cholesterol side chain cleavage enzyme (P450scc) converts it into pregnenolide to initiate steroid hormone synthesis [30,31]. Throughout this process, FDX1 facilitates electron transfer from reduced nicotinamide adenine dinucleotide phosphate to P450scc via FDX reductase, thereby sustaining P450scc activity [32]. Notably, our study findings indicate predominant FDX1 expression in human cumulus cells (CCs), which correlates with germ cell quality. Furthermore, epidermal growth factor and its receptors on cumulus granulosa cells play a pivotal role in promoting steroid hormone growth by modulating Star activity. These hormones, in turn, facilitate oocyte maturation through corresponding receptors [33]. 

In our study, our primary achievement is indeed the identification of FDX1 as a potential biomarker of ovarian aging. However, our research extends beyond this by elucidating the effects of known nutritional supplements, such as DHEA, CoQ10, and T3, on FDX1 gene expression and its impact on cellular energy metabolism pathways. While these nutrients have been previously investigated for their role in improving fertility, our study provides novel insights into their specific mechanisms of action, particularly in relation to FDX1 and energy metabolism pathways within cumulus cells. By demonstrating that these supplements can enhance FDX1 expression and modulate cellular energy metabolism, we contribute to a deeper understanding of their therapeutic potential in improving ovarian function and fertility outcomes. This finding represents a significant advancement in the field, as it highlights the specific molecular pathways through which these nutrients exert their beneficial effects, thereby enriching our knowledge and paving the way for more targeted therapeutic interventions in the future.

Our research team has dedicated extensive efforts to unraveling the molecular intricacies governing ovarian germ cells, with a particular focus on identifying nutritional supplements and potential biomarkers capable of enhancing fertility outcomes in patients [34]. Through our investigation, we have uncovered compelling evidence suggesting that the administration of nutritional supplements can orchestrate a profound reprogramming of metabolic pathways within aging germ cells, thereby instigating the activation of mitochondrial aerobic respiration [35,36,37]. Our results strongly indicate a potential association between this phenomenon and the mitochondrial metal metabolism gene FDX1, similar to our previous findings. In earlier research, we identified the essential metabolic enzyme PGAM5 as a reliable biological indicator for diagnosing aging ovaries, reinforcing the consistency of our current findings with past studies [19]. This reduction in metabolic gene expression can impact cellular pathways, including those involved in programmed cell death. Furthermore, our study is consistent with the prior literature, showing the dual protective effects of nutritional supplements on human granulosa cells. These supplements not only slow down apoptosis and necroptosis but also create a protective environment for cumulus cells through metabolic reprogramming [38].

Nutritional supplements are widely recognized for their ability to modulate mitochondrial metabolism, playing a crucial role in cellular energy production and homeostasis. However, the specific influence of these supplements on the regulation of FDX1 expression remains relatively unexplored in the scientific literature. FDX1, a key gene involved in metal metabolism within mitochondria, holds significant importance in maintaining mitochondrial function and energy metabolism. Dysregulation of FDX1 has been implicated in disrupting mitochondrial activity, leading to the overproduction of reactive oxygen species (ROS) and perturbation of signaling pathways critical for angiogenesis. The potential impact of nutritional supplements on FDX1 function presents an intriguing avenue for research, as it may offer novel insights into the mechanisms underlying ovarian aging. By targeting FDX1 expression, these supplements may exert a regulatory effect on mitochondrial metabolism, thereby mitigating oxidative stress and preserving cellular health in aging ovaries. Furthermore, our prior utilization of coenzyme Q10 (Q10) to mitigate oxidative stress-induced cell death in aging, or oxeiptosis, underscores the multifaceted benefits of nutritional supplementation [8,39,40]. It has been elucidated that DHEA activates CREB1, a pivotal transcription factor governing energy metabolism, thereby modulating downstream gene expression involved in biosynthesis pathways like AMPK, SIRT1, and PGC1α [37]. Consequently, DHEA may exert its regulatory influence on transcription factors implicated in cellular energy metabolism pathways, thereby mitigating programmed cell death associated with aging, such as mitochondrial autophagy, necroptosis, apoptosis, and ferroptosis, as delineated in our study [41]. However, it is imperative to acknowledge the limitations of our study, particularly the relatively small sample size of 30 patients, necessitating cautious interpretation of the data.

## 5. Conclusions

This study introduces an innovative multi-omics strategy to investigate the involvement of FDX1, a crucial gene in mitochondrial metal metabolism, in the modulation of ovarian aging. Seventy infertile patients showing signs of ovarian aging participated in the study, were provided nutritional supplements, and had cumulus cells analyzed. The findings indicated a notable rise in the transcriptional activity of FDX1 in aging cumulus cells following nutritional supplementation. This elevation was concomitant with alterations in the glycolytic pathway and heightened expression of genes associated with the TCA cycle. These enhancements in cellular energy metabolism among senescent cells contribute to the amelioration of egg quality in infertile patients.

## Figures and Tables

**Table 1:** Basic characteristics of patients in the aging and aging/nutri. groups.

Parameters	Aging (n = 40)	Aging/Nutri. (n = 30)
Age (years)	39.4 ± 4.1	39.9 ± 3.2
BMI (kg/m^2^)	24.6 ± 3.5	24.8 ± 3.8
Duration of infertility (years)	3.1 ± 1.7	2.7 ± 2.6
Previous IVF failure (n)	1.4 ± 2.3	1.8 ± 1.2
Types of infertility n (%)		
Primary infertility	17/40 (42.5%)	16/30 (53.3%)
Secondary infertility	23/40 (57.5%)	14/30 (46.7%)
Basal FSH (IU/L)	5.1 ± 5.4	5.5 ± 3.6
Basal E2 (pg/mL)	109.8 ± 72.8	103.8 ± 77.2
Basal LH (IU/L)	6.4 ± 6.8	6.2 ± 5.8

IVF, in vitro fertilization; FSH, follicle stimulation hormone; E2, Estradiol; LH, luteinizing hormone.

**Table 2:** Cycle characteristics and pregnancy outcome in the aging and aging/nutri. groups.

Parameters	Aging (n = 40)	Aging/Nutri. (n = 30)
Stimulation duration (days)	10.7 ± 2.8	10.6 ± 1.4
No. of oocytes retrieved (n)	6.5 ± 3.9	14.2 ± 6.4 **
No. of metaphase II oocytes (n)	5.4 ± 3.1	11.6 ± 5.2 ***
Maturation rate (%)	79.2 ± 18.6	82.4 ± 18.2
No. of fertilized oocytes (n)	4.6 ± 3.2	8.9 ± 4.1 **
Fertilization rate (%)	85.2 ± 20.7	84.6 ± 17.4
No. of Day 3 embryos (n)	4.4 ± 3.7	8.6 ± 4.2 **
No. of top-quality D3 embryos (n)	2.1 ± 1.7	3.2 ± 2.8 **

** *p* < 0.01, *** *p* < 0.001.

## Data Availability

No new data were created or analyzed in this study. Data sharing is not applicable to this article.
